# Effects of Aerobic Exercise on Markers of Brain Injury in Methamphetamine-Dependent Individuals: A Randomized Controlled Trial

**DOI:** 10.3390/brainsci12111521

**Published:** 2022-11-10

**Authors:** Zhicheng Zhu, Jisheng Xu, Yu Jin, Lu Wang, Xue Li

**Affiliations:** 1 School of Sports Medicine and Health, Chengdu Sport University, Chengdu 610041, China; 2 Sichuan Ziyang Compulsory Isolation Detoxification Institute, Ziyang 641300, China

**Keywords:** aerobic exercise, methamphetamine, neurons, blood–brain barrier

## Abstract

Objective: Drug dependence has become a major global public health problem. This study aimed to investigate the effects of moderate-intensity aerobic exercise on the blood–brain barrier and neurological damage in methamphetamine (MA)-dependent individuals. Methods: MA-dependent individuals (all males) were recruited and randomly divided into MA exercise group (MAE) and MA control group (MAC) by using random number table method. The MAE group underwent 12 weeks of moderate-intensity aerobic exercise, and the MAC group underwent conventional detoxification. The Neurofilament light chain (NfL), S100 calcium binding protein b (S100b), and Neuron-Specific Enolase (NSE) levels in the blood of MA-dependent individuals were measured before and after the exercise intervention. Results: After the exercise intervention was implemented, the amount of change in NfL in the plasma of the MAE (1.75 ± 1.40) group was significantly different from that of the MAC (0.60 ± 1.21) group (*p* < 0.01); the amount of change in NSE in the serum of the MAE [−1.51 (−3.99~0.31)] group was significantly different from that of the MAC [0.03 (−1.18~1.16)] group (*p* < 0.05); and the amount of change in S100b in the serum of the MAE [0.66 (0.40~0.95)] group was not significantly different from that of the MAC (0.60 (0.21~1.04)) group (*p* > 0.05). Conclusion: This study showed that 12 weeks of moderate-intensity aerobic exercise treatment significantly promoted the recovery of blood–brain barrier and neurological damage in MA-dependent patients compared with conventional withdrawal.

## 1. Introduction

According to the *World Drug Report 2021*, approximately 275 million people worldwide use drugs, representing approximately 3.62% of the total global population. The number of people using drugs increased by 22% between 2010 and 2019, and based on population trends, projections indicate that the number of people using drugs will increase by 11% globally by 2030 [1]. The toxic effects of methamphetamine (MA) intake include endoplasmic reticulum stress, mitochondrial apoptosis, and dopaminergic and 5-hydroxytryptaminergic impairment [2,3]. Recent studies have shown that MA-mediated neurotoxic effects lead to structural and functional impairment of the blood–brain barrier [4,5], which is an important structure for the regulation of micro-environmental homeostasis in the brain. Its restriction of the free movement of substances between the blood and the central nervous system ensures the homeostasis of the brain microcirculation and provides the necessary environment for neuronal activity and the work of the central nervous system [6]. Disruption of the blood–brain barrier, one of the most prominent events of MA neurotoxicity, exacerbates the penetration of inflammatory factors and viral and microbial pathogens into the central nervous system, further aggravating the neurotoxic effects of MA and leading to neurological damage in MA-dependent individuals [7]. Functionally, it exacerbates deficits and impairments in inhibitory control, learning memory, and social cognition in MA-dependent individuals; especially in inhibitory control, the impairment of which could lead to compulsive drug use and overdose behavior in MA-dependent individuals, resulting in a vicious cycle [8,9].

In recent years, aerobic exercise has shown unique effects as an intervention in the treatment of MA dependence [10]. Studies have shown that exercise could restore or even reverse the damage caused by drug abuse, and enhance physical fitness and improve the psychological condition of MA-dependent individuals; preclinical studies have also shown that exercise intervention could increase neurogenesis in rat hippocampus, promote glial cell production in the medial prefrontal lobe, and reduce MA-induced oxidative stress in mouse brain vasculature by increasing the antioxidant capacity of capillaries, thereby preventing blood–brain barrier disruption [7,11,12,13,14,15,16]. Numerous studies have shown that Neuron-Specific Enolase (NSE), S100 calcium binding protein b (S100b), and Neurofilament light chain (NfL), can be used as markers of blood–brain barrier damage and neuronal axonal damage [17,18,19,20,21,22]. By contrast, these have not been clinically studied to show the effects of exercise interventions on them, and the protective effects of physical activity on the brain of MA-dependent individuals are mainly based on animal studies. Given the protective effects of pre-exercise on the blood–brain barrier and brain microvasculature, the present study hypothesized that moderate-intensity aerobic exercise could further promote recovery of the blood–brain barrier, brain neurons, and inhibitory control in MA-dependent individuals relative to conventional detoxification.

## 2. Materials and Methods

### 2.1. Experimental Design

This study is a randomized, investigator- and outcome-indicator-blinded trial with subjects recruited from April 2021 to June 2021, who signed an informed consent form (see https://www.chictr.org.cn/ for details, accessed on 6 March 2021) prior to the start of the study. The study strictly adhered to the Helgiacin Declaration, and the experimental protocol was approved by the Ethics Committee of the Chengdu Sports Institute and registered with the China Clinical Trials Center (ChiCTR: 2200055348). MA-dependent individuals were screened strictly on the basis of meeting the inclusion and exclusion criteria. They were randomly divided into MA exercise group (MAE) and MA control group (MAC) in a 1:1 ratio by using random number table method. The MAC group underwent conventional educational detoxification, including educational correction and group psychological counseling, while the MAE group received 12 weeks of moderate-intensity aerobic exercise on the basis of conventional education. Due to the specificity of the intervention, the grouping results were not blinded to MA dependence but hidden from the raters of outcome indicators and the investigator. This study was reported in strict compliance with the CONSORT statement [23].

### 2.2. Experimental Design

Based on the results of the pre-experiment, we used STATA 16.0 for the sample size calculation. With α = 0.05 and power = 0.8, assuming equal numbers of MAC and MAE, at least 25 people are needed in each group, and considering a 15% shedding rate, at least 30 people are needed in each group.

### 2.3. Study Subjects

#### 2.3.1. Recruitment and Grouping of Study Subjects

**Figure 1:**
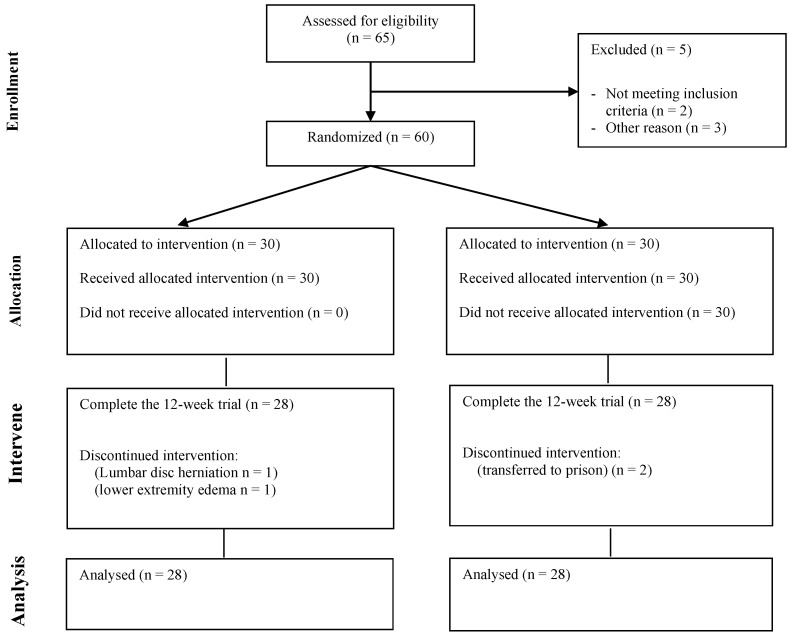
Flow chart of subject recruitment.

This study was conducted within the Ziyang Compulsory Isolation Drug Treatment Center in Sichuan Province, after obtaining approval from the Ethics Committee of Chengdu Sports Institute. Screening was performed in accordance with the inclusion and exclusion criteria, and an informed consent was signed for inclusion in this study. A total of 65 MA-dependent subjects were recruited. Among then, two subjects did not meet the criteria for MA dependence in DSM-V, and one subject had mixed MA and cocaine use. In addition, one case of each suffering from lumbar disc herniation and gout were excluded. A total of 60 MA-dependent subjects were included. During the intervention period, one subject in the MAE group stopped exercising due to a lumbar disc herniation, and another subject stopped exercising due to swelling of the lower extremities. Two subjects in the MAC group were transferred to prison due to involvement in other judicial cases, resulting in attrition (Figure 1).

#### 2.3.2. Inclusion and Exclusion Criteria

The inclusion criteria were as follows: (1) aged 18–45 years; (2) meeting the criteria for MA dependence in the *Diagnostic and Statistical Manual of Mental Disorders, Fifth Edition* (DSM-V); (3) having elementary school education or above; (4) having undergone exercise risk assessment and being qualified for enrollment; (5) being able to guarantee more than 6 months of recovery time; (6) voluntarily participating and signing an informed consent form; (7) showing positive MA urine test within the last 1 year. Subjects were excluded if they (1) had infectious diseases such as hepatitis, HIV, and serious untreated trauma; (2) had recent neurological injuries, such as cranio-cerebral injury or spinal cord injury or suffered from serious mental illness; (3) suffered from serious organic diseases; (4) had other illicit drug dependence in addition to MA dependence. Subjects were considered to be detached if they were (1) unable to continue to participate in the exercise for medical reasons; (2) subjectively did not want to continue to participate in this experiment; (3) required transfer to detention for other cases.

### 2.4. Exercise Intervention Program

The exercise group intervention program implemented moderate-intensity aerobic exercise in accordance with the American College of Sports Medicine guidelines. It was conducted in the Ziyang Compulsory Isolation Drug Rehabilitation Center for 12 weeks, from 9:30 to 10:30 a.m. every Monday through Friday, for 1 h each time (including 10 min of warm-up training, 30 min of aerobic training, and 20 min of stretching), with training including jogging and power biking. Heart rate was monitored during exercise by using a Polar meter, and heart rate was controlled at 65–75% HRmax (HRmax = 206.9 − 0.67 × age). The control group underwent conventional educational detoxification, including educational correction and group psychological counseling.

### 2.5. Exercise Intervention Program

Blood was collected before the exercise intervention and on the following day after the exercise from 7 a.m. to 8 a.m. Fasting venous blood was collected from the subjects. Venous blood was collected from the subjects by using a normal blood collection tube (5 mL, left to stand for 30 min at room temperature (25 °C), and centrifuged (2000 rpm) for 5–10 min. The supernatant was transferred to a 1.5 mL EP tube and immediately stored in a −80 °C refrigerator until analysis. Venous blood was collected from the subjects by using EDTA anticoagulation tubes (2 mL), allowed to stand at room temperature (25 °C) for 30 min, centrifuged (2000 rpm) for 5–10 min. The supernatant was transferred to a 1.5 mL EP tube and immediately stored in a −80 °C refrigerator until analysis. For NfL measurements, the Simoa NF-Light Advantage kit from Quanterix was used on a fully automated instrument, HD-1 Analyzer (Quanterix). Serum NSE and S100b concentrations were determined by ELISA using kits purchased from Human NSE and Human S-100b manufactured by Shanghai Zhoucai Biologicals.

### 2.6. Statistical Analysis

Data were double-entered and analyzed by two researchers to ensure data quality. All data were statistically analyzed using SPSS 26.0 and plotted using GraphPad Prism 9.0 with a test level of 0.05 and a confidence level of 95%. Due to sample attrition, the full analysis set was used to analyze all data in this study. For blood indicators NfL, NSE and S100b, the difference between the two groups before and after the exercise intervention (pre-exercise–post-exercise) was used for comparison to determine the effect of the exercise intervention. For continuous type data, two independent samples *t*-test was used for analysis if the data conformed to normal distribution and chi-square; otherwise, Mann–Whitney *U* test was used. For ordered and unordered categorical variables, Mann–Whitney-U test and chi-square test were used, respectively. Due to missing samples, this study was filled using multiple interpolation, and the differences between the two groups of data were compared by calculating the before and after differences to assess the robustness of the above findings.

## 3. Results

### 3.1. Baseline Conditions of Subjects

General data were collected on the included study subjects, including name, age, duration of drug use, years of drug use, type of drug use, and drug dose, and all data were collected from April 2021 to November 2021. The baseline profiles of the subjects in both groups are shown in Table 1. The results showed no significance in age (*p* = 0.39), height (*p* = 0.69), weight (*p* = 0.14), degree of drug dependence (*p* = 0.84), education (*p* = 0.63), duration of drug use (*p* = 0.98), amount per intake (*p* = 0.63), and number of compulsory isolation (*p* = 0.51, *p* > 0.05), and they were comparable at baseline.

### 3.2. Changes in Serum NSE and S100b among Subjects before and after Aerobic Exercise Intervention

#### 3.2.1. Serum NSE and S100b Levels among Subjects before Aerobic Exercise Intervention

**Figure 2:**
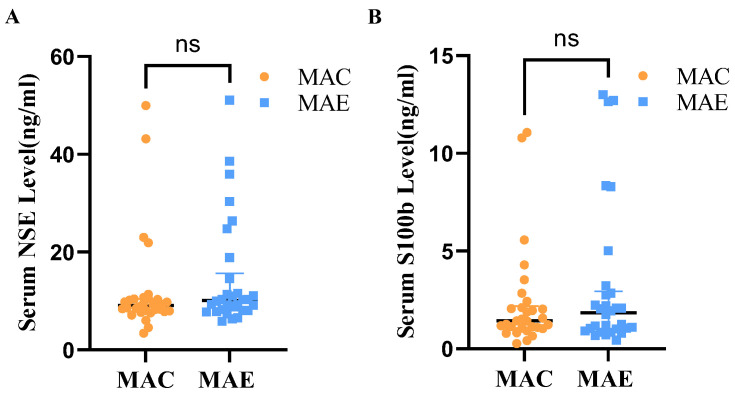
Serum NSE and S100b levels in MAE and MAC groups. (**A**): Serum NSE levels in the MAC and MAE groups; (**B**): Serum S100b levels in the MAC and MAE groups. ns: There was no significant difference in Serum NSE and S100b in the MAE group compared with the MAC group.

Mann–Whitney *U* test was used to determine whether the serum NSE levels differ between the MAE and MAC groups (Table 2, Figure 2). The shape of the distribution of NSE levels in the serum of the two groups was basically the same as judged by the histogram, and the Mann–Whitney results showed that the medians of the MAC and MAE groups were 9.08 and 10.12, respectively, without statistically significant difference (U = 366.000, *p* = 0.21).

Mann–Whitney *U* test was also used to determine whether the serum S100b levels differ between the MAE and MAC groups (Table 2 and Figure 2). The shape of the distribution of NSE levels in the serum of the two groups was basically the same as judged by the histogram, and the Mann–Whitney results showed that the medians of the MAC and MAE groups were 1.43 and 1.85, respectively, without statistically significant difference (U = 415.000, *p* = 0.61).

#### 3.2.2. Serum NSE and S100b Levels among Subjects before and after Aerobic Exercise Intervention

**Figure 3:**
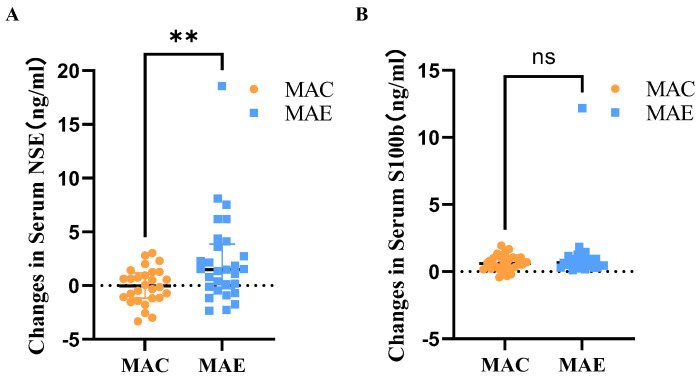
Amount of change in serum NSE and S100b in MAC and MAE groups before and after exercise intervention. (**A**): Amount of change in Serum NSE in MAC and MAE groups before and after exercise intervention.; (**B**): Amount of change in Serum S100b in MAC and MAE groups before and after exercise intervention. **: There was a significant difference in the amount of change in serum S100b before and after the exercise intervention in the MAE group compared to the MAC group; ns: Compared with the MAC group, there was no significant difference in the amount of change in serum NSE in the MAE group before and after the exercise intervention.

After 12 weeks of exercise intervention, the serum NSE levels in the MAC and MAE groups were 12.51 ± 10.67 and 12.55 ± 11.02, respectively, and the amounts of change before and after the exercise intervention were 0.04 ± 1.65 and 2.29 ± 4.17, respectively. The Mann–Whitney *U* test was used to determine the between-group differences in the amount of change (Table 3 and Figure 3). The shape of the distribution of the amounts of change between the two groups was not consistent according to the histogram, and the Mann–Whitney results showed that the mean ranks were 23.21 in the MAC group and 34.59 in the MAE group, with statistically significant difference between-group (U = 244.000, *p* = 0.01).

After 12 weeks of exercise intervention, the serum S100b levels in the MAC and MAE groups were 1.77 ± 2.67 and 2.13 ± 3.36, respectively, and the amounts of change before and after the exercise intervention were 0.67 ± 0.59 and 1.11 ± 2.17, respectively. The Mann–Whitney *U* test was used to determine the between-group differences in the amount of change (Table 3 and Figure 3). The shape of the distribution of the amounts of change between the two groups was not consistent according to the histogram, and the Mann–Whitney results showed that the mean ranks were 27.88 in the MAC group and 30.09 in the MAE group, with no statistically significant difference between-group (U = 374.000, *p* = 0.62).

### 3.3. Plasma NfL Changes in Subjects before and after Aerobic Exercise Intervention

#### 3.3.1. Plasma NfL Levels in Subjects before Aerobic Exercise Intervention

**Figure 4:**
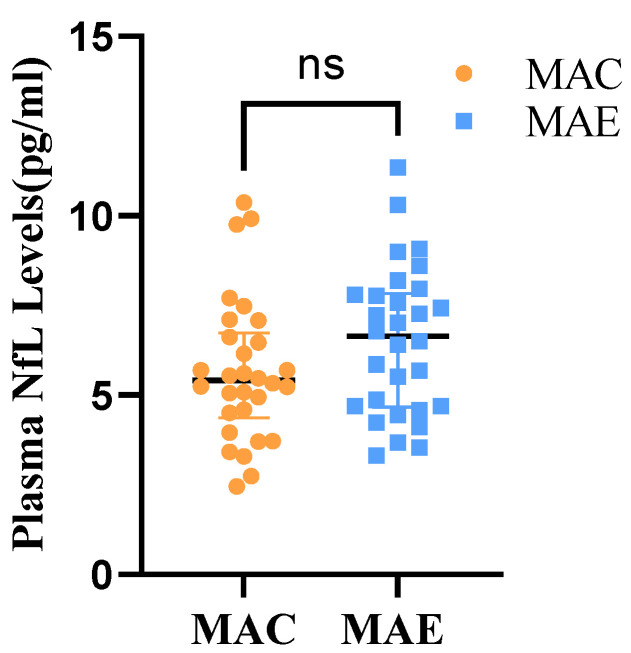
Plasma NfL levels in MAE group and MAC group. ns: There was no significant difference in Plasma NfL in the MAE group compared with the MAC group.

A two independent samples *t*-test was used to determine whether the plasma NfL levels differ between the MAE and MAC groups (Table 4 and Figure 4). The study data did not have significant outliers and conformed to a normal distribution within both groups. The results showed that the plasma NfL levels were 5.66 ± 1.99 in the MAC group and 6.52 ± 2.07 in the MAE group. Moreover, t = −1.630 (*p* > 0.05), indicating no statistical difference in the plasma NfL between the MAE and MAC groups before the exercise intervention.

#### 3.3.2. Plasma NfL Differences in Subjects before and after Exercise Intervention

**Figure 5:**
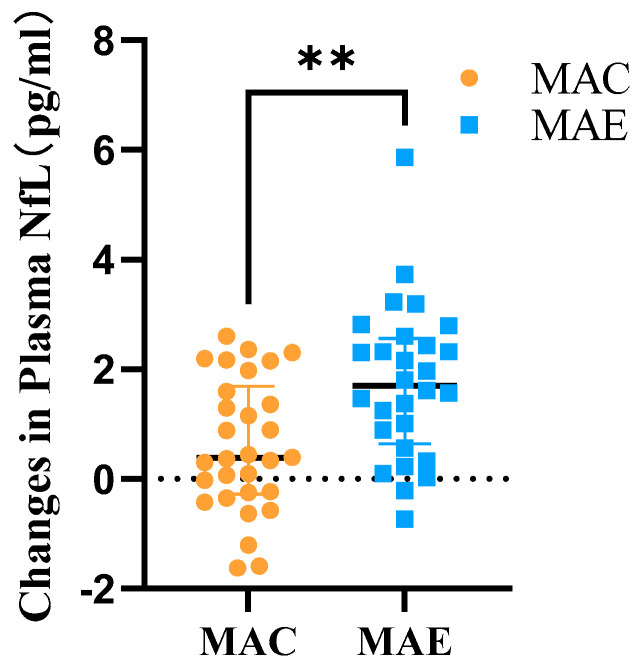
Amount of change in plasma NfL in the MAC and MAE groups after exercise intervention. **: There was a significant difference in the amount of change in Plasma NfL before and after the exercise intervention in the MAE group compared to the MAC group.

After 12 weeks of exercise intervention, the plasma NfL levels in the MAC and MAE groups were 5.06 ± 1.74 and 4.85 ± 1.70, respectively, and the amounts of change in these levels before and after the exercise intervention were 0.60 ± 1.21 and 1.75 ± 1.40, respectively. The two independent samples *t*-test was used to determine the between-group differences in the amount of change (Table 5 and Figure 5). The study data did not have significant outliers and conformed to a normal distribution within both groups, while the variances were flush. The results showed t = −3.348 (*p* < 0.05), with statistically significant difference between-group.

### 3.4. Sensitivity Analysis

Multiple interpolation method was used to fill in the missing NfL data. The results showed that the plasma NfL level in the MAC group was 5.06 ± 1.72 after exercise intervention, and the difference before and after the intervention was 0.60 ± 1.19. Meanwhile, the plasma NfL level in the MAE group was 4.77 ± 1.69, and the difference before and after the exercise intervention was 1.76 ± 1.4. The Mann–Whitney *U* test was used to determine the between-group differences in the amount of change (Table 6). The shape of the distribution of the amounts of change between the two groups was not consistent according to the histogram, and the Mann–Whitney results showed that the mean ranks were 138.17 in the MAC group and 221.30 in the MAE group, with statistically significant difference between-group (U = 8580.000, *p* = 0.001).

Multiple interpolation was also used to fill in the missing NSE data. The results revealed that the serum NSE level in the MAC group was 12.24 ± 10.32 after exercise intervention, with a difference of −0.15 ± 1.77 before and after the intervention. Meanwhile, the serum NSE level in the MAE group was 12.33 ± 10.79, with a difference of 2.32 ± 4.07 before and after the exercise intervention. The Mann–Whitney *U* test was used to determine the between-group differences in the amount of change (Table 6). The shape of the distribution of the amounts of change between the two groups was not consistent according to the histogram, and the Mann–Whitney results showed that the mean ranks were 140.99 in the MAC group and 216.79 in the MAE group, with statistically significant difference between-group (U = 9166.000, *p* = 0.001).

Multiple interpolation was also used to fill in the missing S100b data. The results showed that the serum S100b level in the MAC group was 1.70 ± 2.58 after exercise intervention, with a difference of 0.63 ± 0.62 before and after the intervention. Meanwhile, the serum NSE level in the MAE group was 2.06 ± 3.30, with a difference of 1.16 ± 2.13 before and after the intervention. The Mann–Whitney *U* test was used to determine the between-group differences in the amount of change (Table 6). The shape of the distribution of the amounts of change between the two groups was not consistent according to the histogram, and the Mann–Whitney results showed that the mean ranks were 168.29 in the MAC group and 189.65 in the MAE group, with no statistically significant difference between-group (U = 14,024.000, *p* = 0.05).

In conclusion, multiple interpolation was used to fill in the missing data, and the results of the analysis based on the dataset obtained from multiple interpolation were consistent with those obtained from the full analysis set. The present results could be considered more reliable.

## 4. Discussion

The results of this study showed that the MAE group had a significant difference in serum NSE after exercise intervention compared with the MAC group, indicating that moderate-intensity aerobic exercise could promote the recovery of the blood–brain barrier in MA-dependent individuals. The plasma NfL between the MAE and MAC groups before and after exercise intervention significantly differed, indicating that moderate-intensity aerobic exercise could promote axonal injury in the central nervous system of MA-dependent individuals’ recovery.

Many studies have shown that exercise could promote physical fitness, improve anxiety symptoms, improve depressive symptoms, improve sleep quality, and reduce illicit drug craving in MA-dependent individuals [12]. Exercise as an important intervention to promote recovery from axonal damage to the blood–brain barrier and central nervous system in MA-dependent individuals appears to be related to exercise as an anti-inflammatory and antioxidant agent, increasing levels of tight junction proteins, promoting changes in neurotransmitters in the brain, and promoting neurogenesis [10].

According to the neuroinflammatory hypothesis, MA intake affects the activity of glial cells. For example, when astrocytes are activated, they release various inflammatory factors, including tumor necrosis factor, causing an increase in the release of inflammatory factors in brain regions, such as hippocampus and striatum, further aggravating the neurotoxicity of MA and promoting damage to the blood–brain barrier [24,25]. By contrast, prolonged moderate-to-intense aerobic exercise induces adaptive mechanisms in the immune system, leading to a decrease in the concentration of interleukin-6 and tumor necrosis factor α in the body while making the concentration of anti-inflammatory factors, such as interleukin-10, increase, thus attenuating the damage of MA on the blood–brain barrier and nerves [26,27]. MA intake could also lead to oxidative stress in endothelial cells, thereby decreasing the level of tight junction proteins and eventually leading to blood–brain barrier dysfunction, and the antioxidant effect of exercise is well known [28]. Animal experiments have shown that exercise prevents MA-induced reduction in glutathione in brain capillaries, i.e., it enhances the antioxidant capacity of brain microvasculature and increases the level of tight junction protein to prevent MA-induced oxidative stress in brain capillaries and blood–brain barrier disruption [7]. Robertson et al. [29] performed an 8-week moderate-intensity aerobic exercise intervention in MA-dependent individuals and examined the changes in brain dopamine receptors by using positron emission tomography. They found that the exercise group showed a significant increase in dopamine D2 and D3 receptor binding potential in the striatum after 8 weeks of intervention. Their results suggested that dopamine deficits in the striatum caused by MA intake could be restored by exercise intervention. Finally, experimental animal studies have shown that exercise also attenuates MA-induced abnormal neurogenesis and promotes glial cell production, further reducing MA-induced neurological damage [11,30].

Although this study showed that exercise could promote the recovery of NSE, S100b, and NfL in MA-dependent individuals, no reference range of normal values has been established clinically. Moreover, after illicit drug withdrawal, their brain regions showed different dynamic changes. For example, the gray matter volumes in the orbitofrontal cortex, caudate nucleus, frontal lobe, parietal lobe, and temporal lobe were smaller in MA-dependent subjects than in healthy subjects at 4–7 days of MA withdrawal [31]. For subjects at 2–3 months of withdrawal, their nucleus accumbens and pallidum were found to be larger than those of healthy controls [32]. At 6 months of withdrawal, MA-dependent subjects had significantly decreased gray matter volumes in the precentral gyrus, caudate nucleus, and cingulate gyrus; at 12 months of withdrawal, the gray matter volumes were larger in the cerebellum and lower in the cingulate gyrus [33]. By contrast, in the present study, the markers of Central Nervous System (CNS) axonal damage and blood–brain barrier in MA-dependent individuals were only examined before and after 12 weeks of exercise intervention. Dynamic monitoring was not performed during the exercise intervention, and no prolonged follow-up investigation was conducted after the intervention. Finally, this study did not investigate aspects of inhibitory control, attention, and executive function in MA-dependent individuals in conjunction with electroencephalogram.

In future studies, sample sizes should be increased to establish reference ranges for normal values of serum NSE and S100b and plasma NfL and investigate inhibitory control, executive function, and attention to further determine the positive effects of aerobic exercise on blood–brain barrier and neurological impairment in MA-dependent individuals. Future work should also pay attention to the markers of blood–brain barrier injury and CNS axonal injury in MA-dependent patients during different time periods after withdrawal, to investigate the mechanisms by which exercise promotes blood–brain barrier recovery and neurological damage recovery.

## 5. Conclusions

In MA-dependent individuals, 12 weeks of moderate-intensity aerobic exercise on top of conventional withdrawal treatment significantly promoted the recovery of blood–brain barrier, brain neurons, and inhibitory control in MA-dependent individuals compared with conventional withdrawal treatment.

## Figures and Tables

**Table 1:** Baseline conditions of subjects in both groups.

	Level	MAC (*n* = 30)	MAE (*n* = 30)	*Z*/*t*	*p*
Age		33.90 ± 4.12	32.97 ± 4.16	0.872	0.387
Height		168.96 ± 6.60	169.26 ± 4.45	−0.394	0.693
Weight		70.54 ± 8.90	68.14 ± 8.51	−1.146	0.143
DSM-V	Mild	7 (23.3%)	8 (26.7%)	−0.205	0.838
	Moderate	13 (43.3%)	10 (33.3%)		
	Severe	10 (33.3%)	12 (40%)		
educational level	primary school	9 (30%)	10 (33.3%)	0.480	0.631
	junior high school	12 (40%)	13 (43.3%)		
	High School and Secondary School	8 (26.7%)	6 (20%)		
	College and above	1 (3.4%)	1 (3.4%)		
drug use time		109.64 ± 43.59	109.97 ± 56.23	−0.024	0.981
C	<0.1 g	7 (23.3%)	4 (13.3%)	0.480	0.631
	0.1–0.3 g	12 (40.0%)	8 (26.7%)		
	0.4–1.0 g	10 (33.3%)	16 (53.3%)		
	>1.0 g	1 (3.3%)	2 (6.7%)		
Number of compulsory isolation and detoxification	The first time	24 (80.0%)	22 (73.3%)	−0.664	0.506
	The second time	6 (20.0%)	7 (23.3%)		
	The third time	0 (0%)	1 (1.7%)		

**Table 2:** Serum NSE and S100b levels in MAE and MAC groups.

Group	MAC (*n* = 30)	MAE (*n* = 30)	*Z*	*p*
NSE (ng/mL)	9.08 (7.93~10.43)	10.12 (8.09~15.65)	−1.242	0.214
S100B (ng/mL)	1.43 (1.05~2.19)	1.85 (1.00~2.94)	−0.517	0.605

**Table 3:** Amount of change in serum NSE and S100b levels and MAC and MAE groups after exercise intervention.

Markers	Group	after Exercise	Difference	*Z*	*p*
NSE (ng/mL)	MAC (*n* = 28)	12.51 ± 10.67	0.03 (−1.18~1.16)	−2.586	0.010
	MAE (*n* = 28)	12.55 ± 11.02	−1.51 (−3.99~0.31) **		
S100B (ng/mL)	MAC (*n* = 28)	1.77 ± 2.67	0.60 (0.21~1.04)	−0.503	0.615
	MAE (*n* = 28)	2.13 ± 3.36	0.66 (0.40~0.95)		

Note: ** The difference in serum NSE of the MAE group was significant compared with that of the MAC group (*p* < 0.01), whereas the difference in S100b was not significant (*p* > 0.05).

**Table 4:** Plasma NfL levels in MAE and MAC groups.

Group	MAC (*n* = 30)	MAE (*n* = 30)	*t*	*p*	*95 CI*
NfL (pg/mL)	5.66 ± 1.99	6.52 ± 2.07	−1.630	0.109	−1.901~1.945

**Table 5:** Plasma NfL levels after exercise intervention and the amount of change in the MAC and MAE groups.

Group	after Exercise	Difference	*t*	*p*
MAC (*n* = 28)	5.06 ± 1.74	0.60 ± 1.21	−3.348	0.001
MAE (*n* = 28)	4.85 ± 1.70	1.75 ± 1.40 ***		

Note: *** Significant plasma NfL difference in the MAE group compared with the MAC group (*p* < 0.001).

**Table 6:** Comparison results of the main outcome indicators after using multiple interpolation method.

Markers	Group	after Exercise	Difference	*Z*	*p*
NfL (pg/mL)	MAC	5.06 ± 1.72	0.38 (−0.24~1.60)	−7.600	0.001
	MAE	4.77 ± 1.69	1.80 (0.81~2.60) ***		
NSE (ng/mL)	MAC	12.24 ± 10.32	−0.14 (−1.24~1.03)	−6.940	0.001
	MAE	12.33 ± 10.79	1.49 (−0.11~4.08) ***		
S100b (ng/mL)	MAC	1.70 ± 2.58	0.55 (0.21~1.02)	−1.956	0.051
	MAE	2.06 ± 3.30	0.66 (0.41~1.05)		

Note: *** The difference in serum NSE of the MAE group was significant compared with that of the MAC group (*p* < 0.001), whereas the difference in S100b was not significant compared with that of the MAC group (*p* > 0.05); the difference in plasma NfL of the MAE group was significant compared with that of the MAC group (*p* < 0.001).

## Data Availability

Not applicable.
